# Treatment with *Ligilactobacillus murinus* lowers blood pressure and intestinal permeability in spontaneously hypertensive rats

**DOI:** 10.1038/s41598-023-42377-7

**Published:** 2023-09-14

**Authors:** Masashi Mukohda, Takanori Yano, Toshiyasu Matsui, Sho Nakamura, Jiro Miyamae, Kensuke Toyama, Ryoji Mitsui, Risuke Mizuno, Hiroshi Ozaki

**Affiliations:** 1 Laboratory of Veterinary Pharmacology, Faculty of Veterinary Medicine, Okayama University of Science, Imabari, Ehime 7948555 Japan; 2 Laboratory of Applied Microbiology, Faculty of Life Science, Okayama University of Science, Okayama, 7000005 Japan; 3 Laboratory of Veterinary Anatomy, Faculty of Veterinary Medicine, Okayama University of Science, Okayama, Ehime 7948555 Japan; 4 Graduate School of Bioagricultural Sciences, Nagoya University, Nagoya, 4648601 Japan; 5 Laboratory of Immunology, Faculty of Veterinary Medicine, Okayama University of Science, Okayama, Ehime 7948555 Japan; 6 Department of Pharmacology, Ehime University Graduate School of Medicine, Toon, Ehime 7910295 Japan

**Keywords:** Hypertension, Hypertension

## Abstract

One feature of hypertension is a microbial imbalance with increased intestinal permeability. In this study, we examined whether an alteration in the microbiota affects blood pressure and intestinal permeability in spontaneously hypertensive rats (SHRs). We performed a 16S metagenome analysis of feces from 10- to 15-week-old SHRs using a synthetic long-read sequencing approach, and found a candidate for the microbiome treatment, *Ligilactobacillus murinus* (*L. murinus*), that was robustly decreased. Oral administration of *L. murinus* to SHRs for 2 weeks significantly inhibited blood pressure elevation and improved endothelium-dependent vasodilation but did not attenuate enhanced vascular contraction in SHR mesenteric arteries. The proximal colon of SHRs exhibited increased intestinal permeability with decreased levels of the tight junction protein claudin 4, morphological changes such as decreased intestinal crypts and elevated TNF-α levels, which was reversed by treatment with *L. murinus*. Consistent with these intestinal phenotypes, plasma lipopolysaccharides levels were elevated in SHR but decreased following *L. murinus* administration. We concluded that oral administration of *L. murinus* to SHRs exerts protective effects on intestinal permeability via restoration of claudin 4 expression and reversal of morphologic disorder, which may improve low-grade endotoxemia and thus reduce development of hypertension via recovery of endothelial vasodilating functions.

## Introduction

Microbiome research has made significant recent progress and generated worldwide focus and attention. Accumulating evidence indicates that the microbiota plays important roles in physiological and pathological events in the host. For instance, the gut microbiome regulates not only host nutrients but also the immune system to protect against infection^1^. A significant relationship exists between gut microbial imbalances and gastrointestinal, metabolic, immune, cardiovascular, psychiatric, and developmental diseases^2, 3^. Additional research is thus required to obtain an in-depth understanding of health and disease in the context of the gut microbiome.

Hypertension is the most common risk factor for cardiovascular disease^4^, and several recent studies have suggested a link between cardiovascular disease and gut dysbiosis^5^. For example, transplantation of fecal microbiota from a hypertensive human donor to germ-free mice was shown to cause blood pressure elevation^6^. Consistent with this observation, angiotensin II-induced hypertension and vascular dysfunction are attenuated in germ-free mice^7^, suggesting that the gut microbiota can cause or accelerate the development of hypertension.

Additionally, in cases of hypertension with dysbiosis, the intestinal permeability is disrupted^8^. Increased intestinal permeability allows allergens and pathogens to penetrate the intestinal barrier and stimulate the immune system^9^. Symptoms associated with increased intestinal permeability are observed in diverse diseases, from inflammatory bowel disorder to infection, cancer, and metabolic disease^10–12^. Accumulating evidence has demonstrated that hypertension, cardiac infarction, and atherosclerosis are also associated with a breakdown in intestinal barrier function^13–15^. Thus, normalization of an imbalance in the gut microbiota and restoration of intestinal permeability are novel research topics in the context of non-gastrointestinal diseases, and such research could lead to the identification of a new class of therapeutic targets for treating cardiovascular disease.

In the present study, we examined whether treatment of spontaneous hypertensive rats (SHRs) with gut microbiota organisms has an effect on blood pressure and intestinal permeability. We found that oral administration of *Ligilactobacillus murinus (L. murinus)* to SHRs for 2 weeks rescued defective vascular endothelial function and lowered blood pressure in the rats and also decreased plasma lipopolysaccharide (LPS) levels. We also found that *L. murinus* administration strengthened the intestinal barrier system in the proximal colon by upregulating tight junction proteins.

## Results

### Ratio of *L. murinus* was decreased in feces of SHRs

Feces from 10- to 15-week-old SHRs and WKY rats were examined by 16S metagenome analysis using a synthetic long-read sequencing LoopSeq approach. The analysis revealed that the rations of two different species of the microbiome, *L. murinus* and *Akkermansia muciniphilla*, were decreased in SHRs compared with WKY rats. The analysis also showed that *Bacteroides caecimuris* and *Bacteroides thetaiotaomicron* were increased in SHRs. We confirmed that one of the characteristic phenotypes, Firmicutes/Bacteroidetes ratio was significantly increased in SHR compared with WKY. These data are shown in Supplementary Fig. 1 online.

### Treatment with *L. murinus* lowered blood pressure and improved vascular endothelial function in SHRs

Because *L. murinus* was relatively easily handled, we determined whether restoration of *L. murinus* affected blood pressure in SHR. We first attempted to isolate *L. murinus* from feces of 10- to 15-week-old WKY rats. Nine strains of 48 candidates were identified as *L. murinus* by PCR using species-specific primer sets (Supplementary Fig. 2). 16S rRNA sequences of the nine strains showed 99.9% identity to the sequence of *L*. *murinus* NBRC 14221 (type strain). These isolated strains were designated *Ligilactobacillus murinus* WO strains (WO9, WO17, WO22, WO28, WO29, WO32, WO39, WO46 and WO48) and divided into three groups by ERIC-PCR. The band patterns of the strains in ERIC-PCR analyses clearly differed from the band pattern of NBRC 14221 (Supplementary Fig. 3).

**Figure 1:**
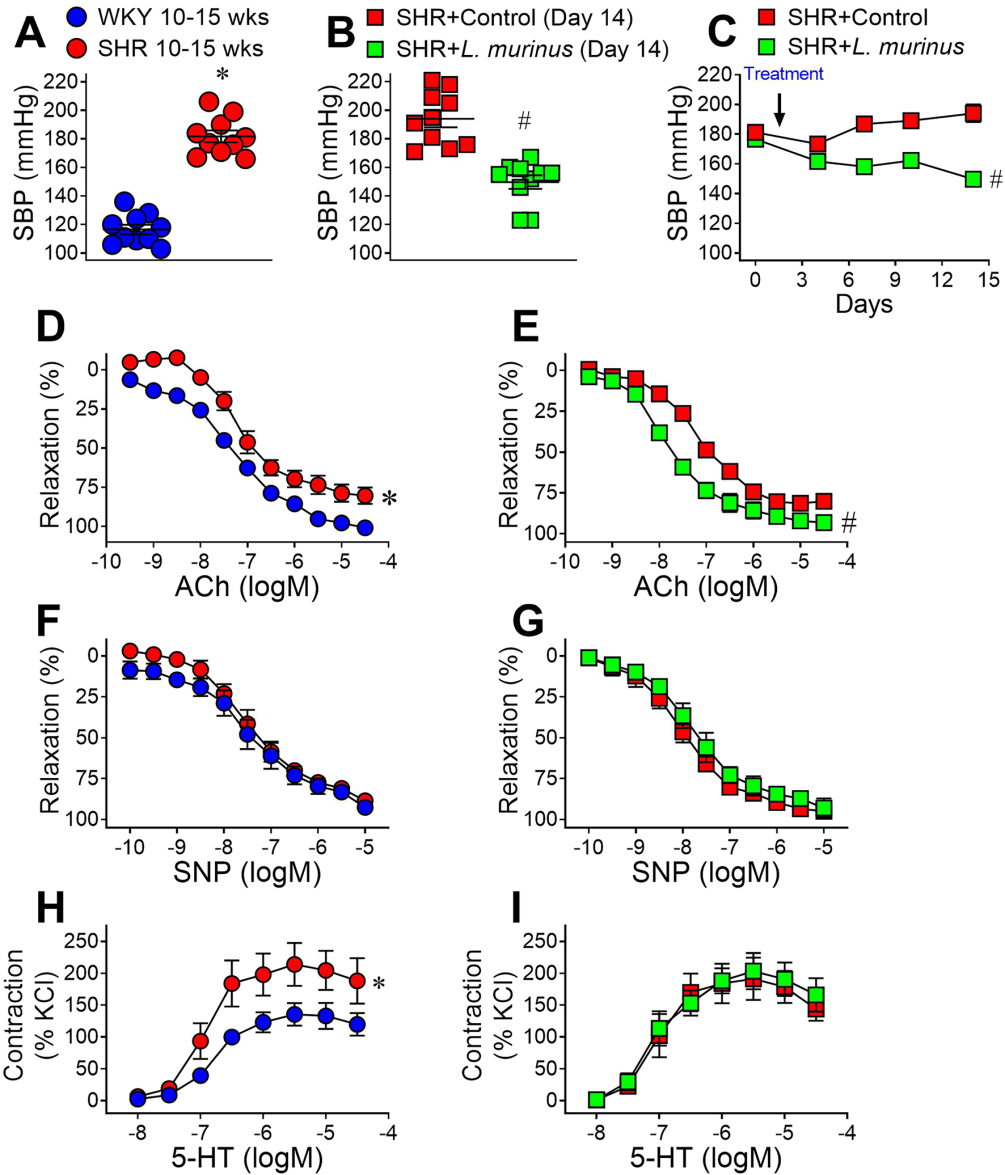
Blood pressure and vascular function in untreated SHRs and SHRs treated with *Ligilactobacillus murinus* (*L. murinus*). (**A**,**B**) Systolic blood pressure (SBP) measured via tail-cuff plethysmography in 10- to 15-week-old WKY rats and SHRs (**A**) or SHRs + Control and SHRs + *L. murinus* (**B**). (**C**) SBP was measured for 14 days after initiation of treatment with Control or *L. murinus* (arrow) in SHRs. (**D**–**I**) Isometric tension experiments were performed using 2nd-branch mesenteric artery in WKY rats, SHRs, SHRs + Control and SHRs + *L. murinus*. Cumulative concentration–response curves for acetylcholine (ACh, 1 nM–30 μM) in mesenteric arteries from WKY rats and SHRs (n = 8 per group) (**D**) and SHRs + Control and SHRs + *L. murinus* (n = 6 per group) (**E**). Cumulative concentration–response curves for sodium nitroprusside (SNP, 0.1 nM–3 μM) in mesenteric arteries from WKY and SHR (n = 8 per group) (**F**) and SHR + Control and SHR + *L. murinus* (n = 6 per group) (**G**). Cumulative concentration–response curves for 5-HT (10 nM–30 μM) in mesenteric arteries from WKY rats and SHRs (n = 8 per group) (**H**) and SHRs + Control and SHRs + *L. murinus* (n = 6 per group) (**I**). All data are mean ± SEM. *P < 0.05 vs. WKY rats or day 0, ^#^*P* < 0.05 vs. SHRs + Control.

We then administered *L. murinus* (as an equal mixture of WO strains, approximately 1 × 10^8^ CFU/mL) to 10- to 15-week-old SHRs orally every day for 2 weeks. Preliminary analyses confirmed an increase in systolic blood pressure (Fig. 1A). Although *L. murinus* orally administered for 2 weeks did not affect the body weight of 10- to 15-week-old SHRs (Supplementary Fig. 4A), the elevation in blood pressure was significantly reversed (Fig. 1B,C and Supplementary Fig. 4B). However, treatment with *L. murinus* did not affect heart rate or the increase in heart weight of SHRs (Supplementary Fig. 4C–G).

We next examined vascular functions in SHRs before and after administration of *L. murinus*. We observed that ACh-induced endothelium-dependent relaxation was impaired in mesenteric arteries of SHRs, and this was significantly reversed by *L. murinus* treatment (Fig. 1D and E). There was no difference in sodium-nitroprusside-induced, endothelium-independent relaxation in any of the groups (Fig. 1F and G). In contrast, vascular contraction in response to 5-HT was increased in SHRs, and this was not affected by *L. murinus* (Fig. 1H and I).

### Increased intestinal permeability in SHR proximal colon was improved by *L. murinus*

**Figure 2:**
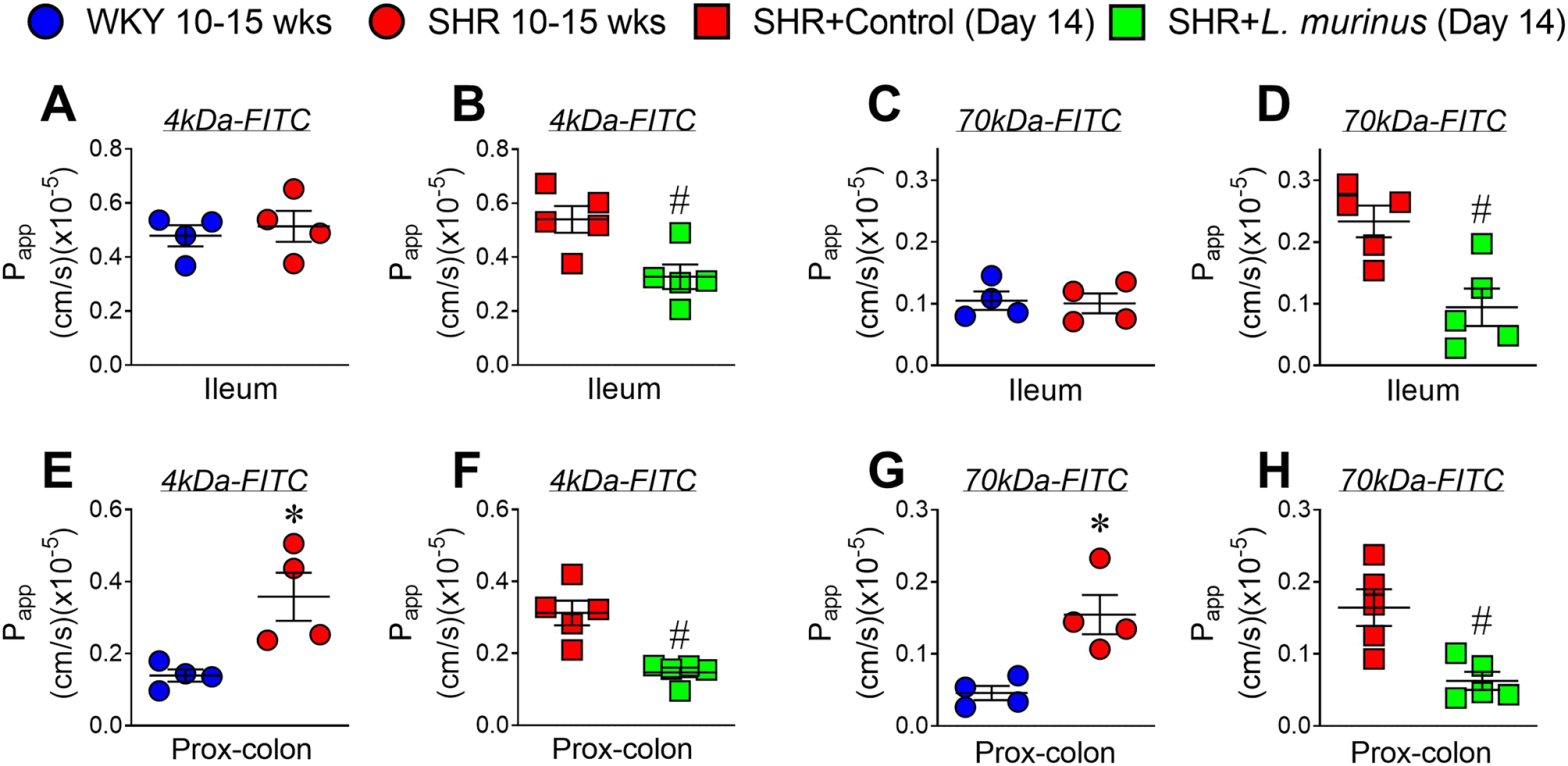
Intestinal permeability in untreated SHRs and SHRs treated with *L. murinus*. (**A**–**D**) Apparent permeability of the ileum of 10- to 15-week-old WKY rats and SHRs (**A**,**C**) (n = 4 per group) or SHRs + Control and SHRs + *L. murinus* (**B**,**D**) (n = 5 per group) was measured using the non-everted gut sac exam with 4-kDa (**A**,**B**) or 70-kDa FITC (**C**,**D**). (**E**–**H**) Apparent permeability of the proximal colon of 10- to 15-week-old WKY rats and SHRs (**E**,**G**) (n = 4 per group) or SHRs + Control and SHRs + *L. murinus* (**F**,**H**) (n = 5 per group) was measured using the non-everted gut sac exam with 4-kDa (**E**,**F**) or 70-kDa FITC (**G**,**H**). All data are mean ± SEM. *P < 0.05 vs. WKY rats, ^#^*P* < 0.05 vs. SHRs + Control.

**Figure 3:**
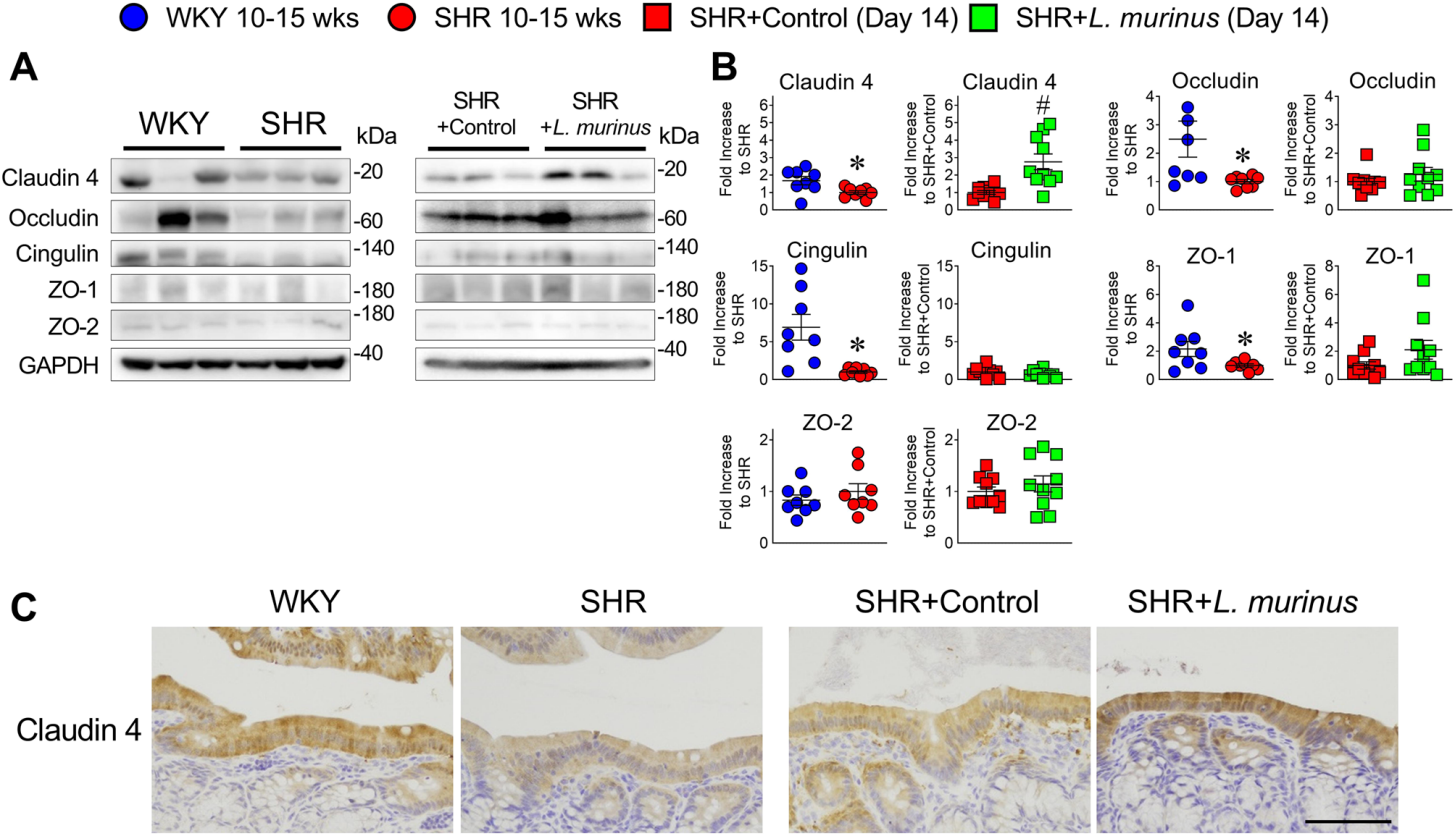
*Tight junction proteins in untreated SHRs and SHRs treated with* L. murinus. (**A**,**B**) Western blotting analysis of indicated proteins (representative of 8–10 experiments) in the proximal colon of 10- to 15-week-old WKY rats and SHRs (n = 8 per group) or SHRs + Control and SHRs + *L. murinus* (n = 10 per group). Representative (**A**) and quantified (**B**) Western blots for claudin 4, occludin, cingulin, ZO-1, and ZO-2. (**C**) Immunostaining analysis of claudin 4 (representative of 4–5). All data are mean ± SEM. *P < 0.05 vs. WKY rats, ^#^*P* < 0.05 vs. SHRs + Control. Scale bar: 50 μm.

Next, we examined whether *L. murinus* affects the integrity of the gut epithelial barrier in SHRs. Adult SHRs (10–15 weeks old) showed altered intestinal permeability in the proximal colon compared with age-matched WKY rats, but no change in permeability of the ileum was observed (Fig. 2A–H). Treatment with *L. murinus* for 2 weeks lowered the permeability of the proximal colon, suggesting that *L. murinus* exerts a protective effect on the intestinal epithelium. We then determined the expression levels of tight junction proteins in SHRs. The expression levels of claudin 4, occludin, cingulin, and ZO-1 were decreased in the proximal colon of SHRs compared with the proximal colon of WKY rats (Fig. 3A and B and Supplementary Figs. 5 and 6). There were no changes in the expression levels of tight junction proteins in the ileum of 10-to 15-week-old SHRs (Supplementary Figs. 7 and 8). Administration of *L. murinus* significantly increased claudin 4 expression but not that of other tight junction proteins. Immunostaining data showed a decrease in claudin 4 expression in the proximal colon intestinal epithelium of SHRs, which was reversed by *L. murinus* (Fig. 3C).

### *L. murinus* ameliorated changes in the intestinal morphology of SHRs

**Figure 4:**
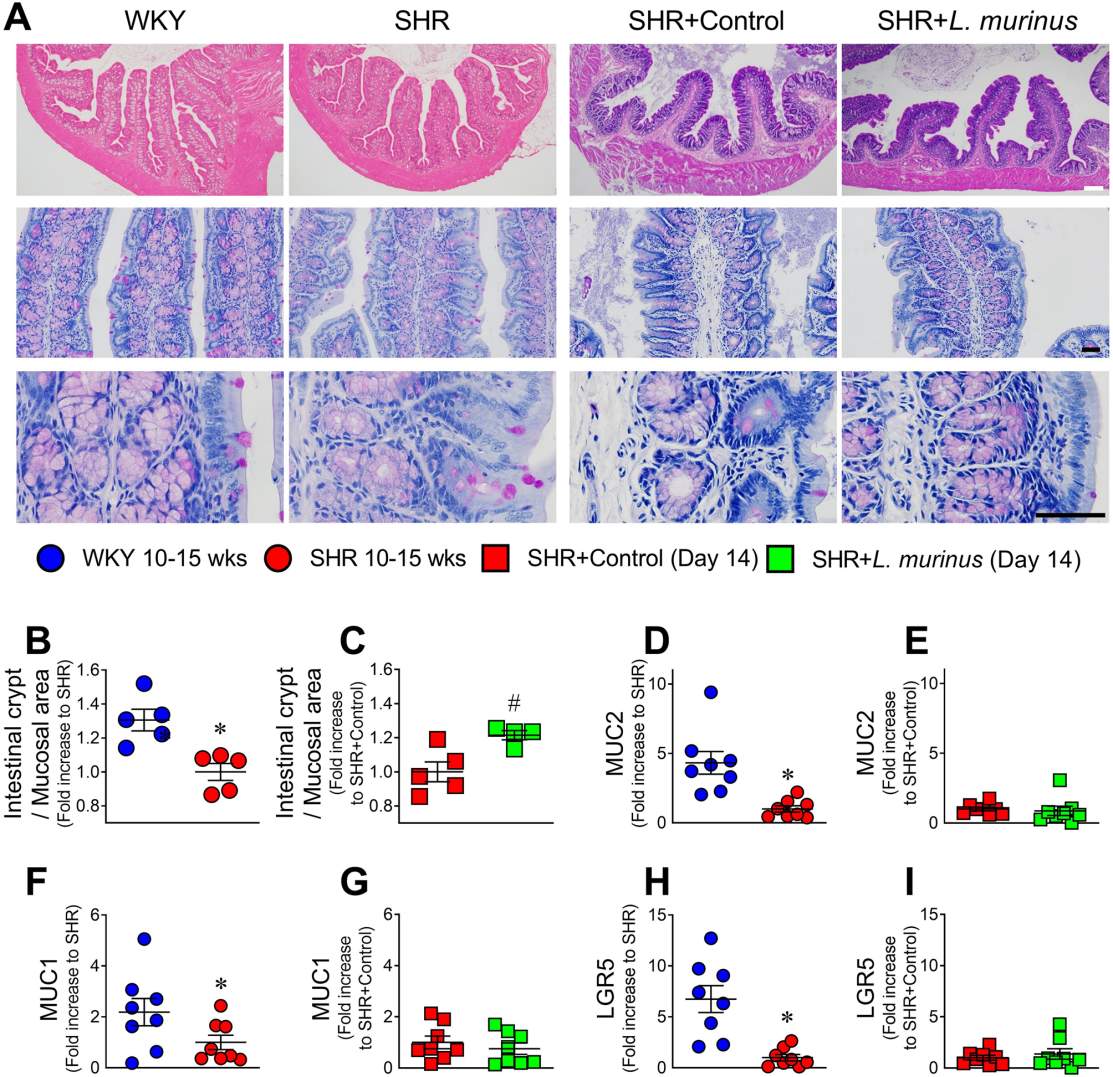
Changes in morphology of the proximal colon of SHRs and SHRs treated with *L. murinus*. (**A**) The proximal colon was sectioned and stained with hematoxylin and eosin or PAS to examine intestinal crypts. (**B**,**C**) Intestinal crypts in proximal colon of 10- to 15-week-old WKY rats and SHRs (**B**) (n = 5 per group) or SHRs + Control and SHRs + *L. murinus* (**C**) (n = 4–5 per group) were measured. Quantitative PCR analysis of mucin 2 (**D**,**E**), mucin 1 (**F**,**G**), and leucine-rich repeat containing G protein-coupled receptor 5 (Lgr5) (**H**,**I**) in proximal colon of 10- to 15-week-old WKY rats and SHRs (n = 8 per group) or SHRs + Control and SHRs + *L. murinus* (n = 8 per group). All data are mean ± SEM. *P < 0.05 vs. WKY rats, ^#^*P* < 0.05 vs. SHRs + Control. White scale: 100 μm, black scale: 25 μm.

We examined the effect of *L. murinus* on intestinal morphology in SHRs. Adult SHRs exhibited a decrease in intestinal crypts in the proximal colon (Fig. 4A and B), but there were no pronounced changes in the duodenum, jejunum, or ileum (Supplementary Fig. 9). The number of goblet cells in the proximal colon was slightly decreased in SHRs compared with WKY rats, but the difference was not significant (Supplementary Fig. 10). Treatment with *L. murinus* restored the intestinal crypts compared with the control group (Fig. 4A–C). Real-time PCR analysis revealed significant decreases in mRNA levels of mucin 2, mucin 1, and leucine-rich repeat containing G protein-coupled receptor (LGR)5 (Fig. 4D–I). In addition, a decreasing trend in telomerase reverse transcriptase (Tert) expression was observed in SHR proximal colon (Supplementary Fig. 11A and B). No change in mucin 4 expression was observed in SHRs (Supplementary Fig. 11C and D). The expression of these genes was not altered by treatment with *L. murinus*.

### Gut inflammation in SHRs was attenuated by *L. murinus*

**Figure 5:**
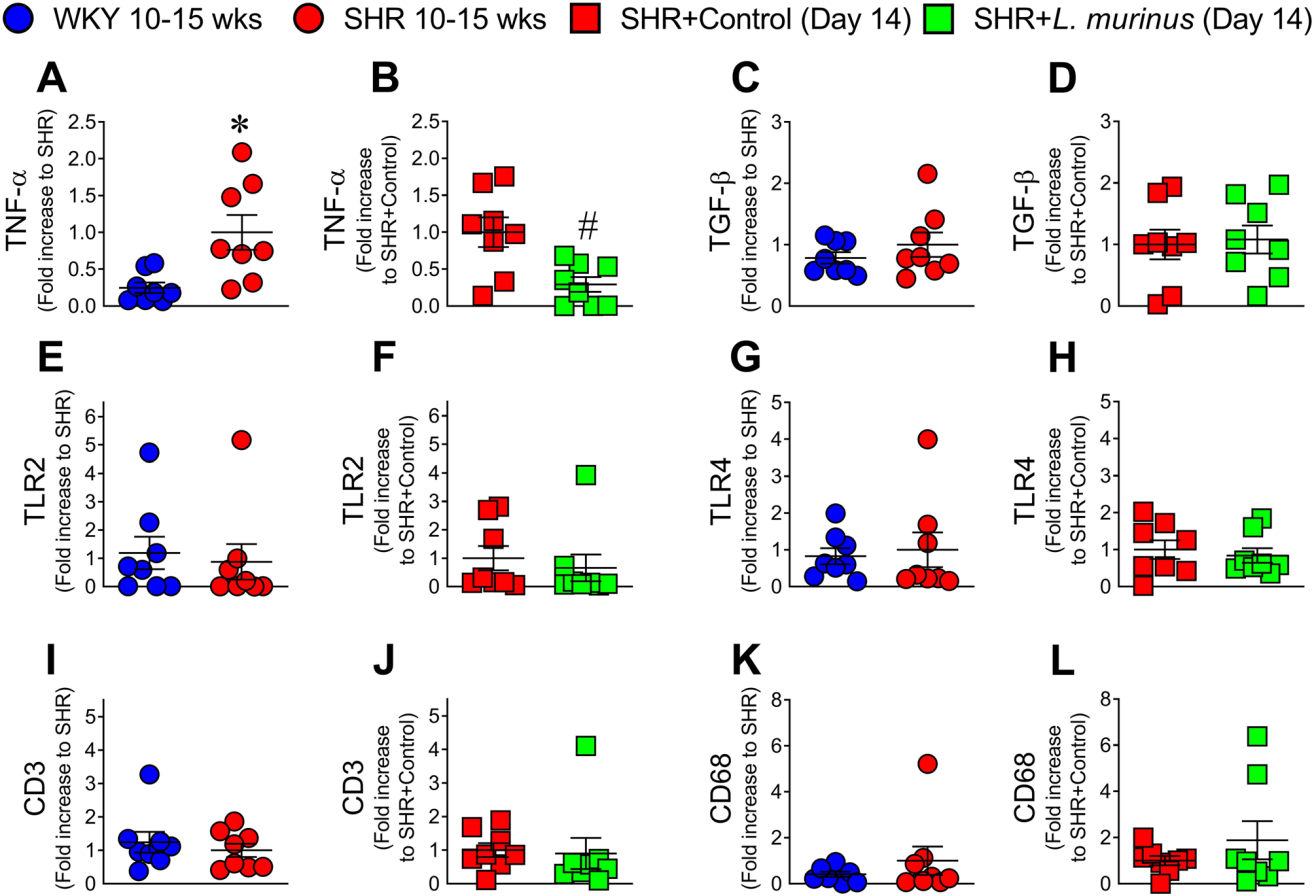
Inflammatory cytokines, toll-like receptors (TLRs), and inflammatory cells in the proximal colon of WKY rats, SHRs, and SHRs treated with *L. murinus*. Quantitative PCR analysis of tumor necrosis factor (TNF)-α (**A**,**B**), transforming growth factor (TGF)-β (**C**,**D**),TLR2 (**E**,**F**), TLR4 (**G**,**H**), CD3 (**I**,**J**), and CD68 (**K**,**L**) in the proximal colon of 10- to 15-week-old WKY rats and SHRs (n = 8 per group) or SHRs + Control and SHRs + *L. murinus* (n = 8 per group). All data are mean ± SEM. *P < 0.05 vs. WKY rats, ^#^*P* < 0.05 vs. SHRs.

We next investigated the effect of *L. murinus* administration on immunologic properties in SHRs. The mRNA level of TNF-α was significantly increased in proximal colon in 10- to 15-week-old SHRs (Fig. 5A). Treatment with *L. murinus* decreased the expression levels of TNF-α (Fig. 5B). In contrast, no differences in the expression of the toll-like receptor 2 (TLR2), TLR4, transforming growth factor β (TGF-β), CD3, and CD68 genes were observed between the WKY and SHR groups (Fig. 5C–L).

T helper 17 cells (Th17) and type 1 helper cells (Th1) are associated with the development of hypertension and cardiovascular target-organ damage, but regulatory T cells (Tregs) exert anti-inflammatory effects^16^. We therefore examined whether these immune cells are involved in gut inflammation in 10- to 15-week-old SHRs using flow cytometry analysis with a specific gating strategy (Supplementary Fig. 12). No changes in total T lymphocytes (CD4+CD45+), Tregs (CD4+FoxP3+), Th1 cells (CD4+INFγ+), or Th17 cells (CD4+IL17+) were observed in SHR lymph nodes (Supplementary Fig. 13A–E) and spleen (Supplementary Fig. 13F–I) compared with age-matched WKY rats.

### Plasma endotoxin levels were decreased by *L. murinus*

**Figure 6:**
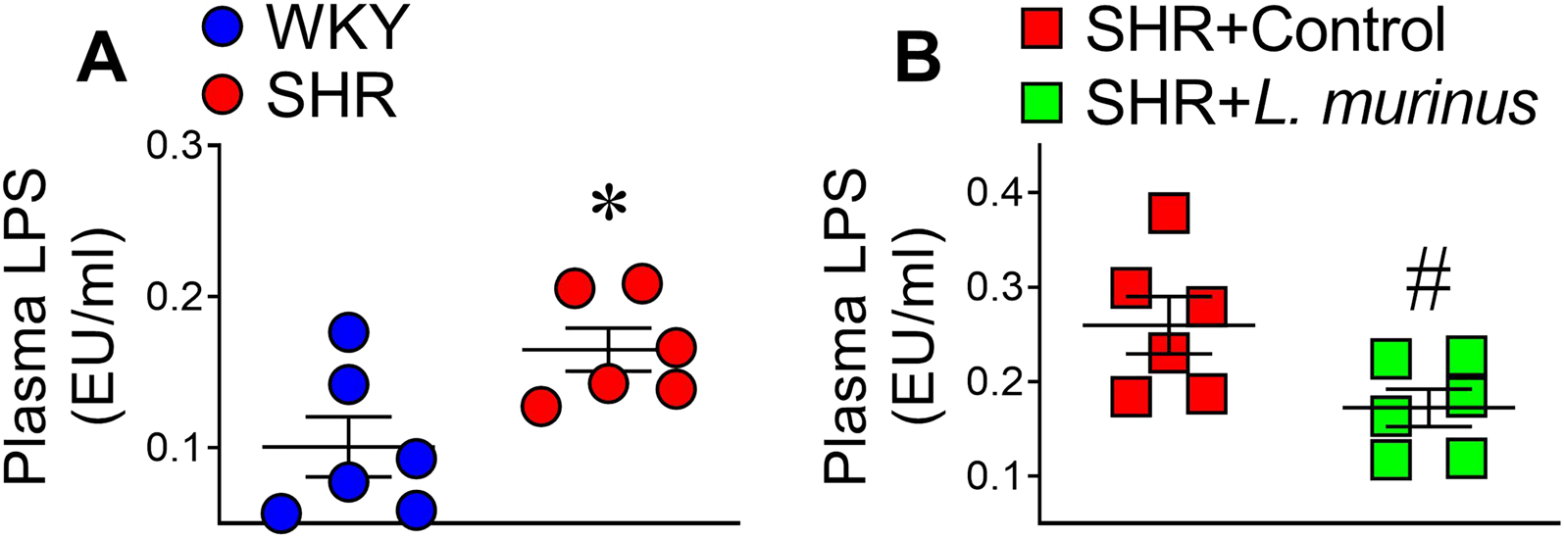
Plasma levels of endotoxin in untreated SHRs and SHRs treated with *L. murinus*. Plasma levels of lipopolysaccharide (LPS) in WKY rats and SHRs (**A**) (n = 6 per group) or SHRs + Control and SHRs + *L. murinus* (**B**) (n = 6 per group). All data are mean ± SEM. *P < 0.05 vs. WKY rats, ^#^*P* < 0.05 vs. SHRs + Control.

LPS exposure induces endothelial dysfunction and vascular inflammation, and human studies have shown that plasma levels of LPS are positively correlated with hypertension^17^. We therefore examined whether *L. murinus* affects LPS levels in SHRs. Plasma levels of LPS were increased in SHRs, and administration of *L. murinus* reversed this increase (Fig. 6).

## Discussion

Accumulating evidence indicates that dysbiosis is strongly associated with the development of cardiovascular diseases, including hypertension. However, the pathophysiologic mechanism by which an imbalance in the gut microbiota causes hypertension remains unclear. In this study, we focused on determining whether treatment to reverse a decrease in specific microbiota affects the gut environment and cardiovascular system in hypertensive rats. The main findings of our study are as follows: (1) two different species of microbiota were markedly decreased in 10- to 15-week-old SHRs; (2) treatment with *L. murinus* for 2 weeks ameliorated the disruption of intestinal barrier function in the proximal colon via increased claudin 4 expression; (3) morphological changes with TNF-α expression in the proximal colon were improved in SHRs treated with *L. murinus*; and (4) vascular dysfunction and blood pressure elevation were reversed with decreasing in plasma levels of endotoxin by *L. murinus* administration. These results support the hypothesis that an imbalance in the gut microbiota is related to the development of hypertension.

Increased intestinal permeability with dysbiosis is observed in several diseases and conditions, including obesity, aging, infection, and cancer^18^. In addition, several reports indicated that hypertensive model exhibited increased intestinal permeability and morphological changes^8, 19, 20^. In this study, we observed an increase in intestinal permeability in the proximal colon with decreased expression of tight-junction proteins (claudin 4, occludin, cingulin, and ZO-1) in hypertensive model rats. It is notable that the increase in intestinal permeability was reversed with increased claudin 4 expression by administration of *L. murinus*. Claudin 4 regulates the paracellular sodium barrier and is expressed in various organs, including the intestine (colon), kidney, and lung^21^. Deletion of claudin 4 reportedly increases susceptibility to lung injury^22^. It is notable that short-chain fatty acids such as acetate reportedly increase claudin 4 expression in intestinal epithelial cells^23^. These findings suggest that metabolites of *L. murinus* increase the expression of claudin 4 in the proximal colon, resulting in improved intestinal barrier function.

Consistent with the change in gut permeability, morphological changes were also observed in hypertensive model rats. The most notable and reproducible phenotype of SHRs was decreased intestinal crypts, which consist of the intestinal epithelium and villi. Crypt-villus tissue, which continuously proliferates, enables the intestine to act as a barrier and also serves as the primary site of nutrient uptake^24^. However, chronic NF-κB activation by TNF-α was shown to reduce intestinal crypts via apoptosis in inflammatory bowel disease^25^. In our study, the proximal colon in SHRs exhibited decreased expression of stem cell markers (LGR5 and Tert) and mucus markers (MUC1, 2, 4). In addition, TNF-α expression was increased in hypertensive model rats, and this was reversed by treatment with *L. murinus*. Pronounced morphological changes were observed in the proximal colon but not the duodenum, jejunum, or ileum of 10- to 15-week-old SHRs.

Host immune cells in the gut interact with the microbiome to regulate systemic immune responses. Numerous studies have shown that cells of the innate and adaptive immune systems play important roles in the development of hypertension^16^. In particular, activation of Th17 and Th1 cells is strongly associated with the development of hypertension^26, 27^, whereas Tregs exert an anti-inflammatory effect on the cardiovascular system via production of IL-10^28^. Unexpectedly, the present study found no significant differences in these immune cells in intestinal lymph nodes and spleen between 10- to 15-week-old SHRs and the relatively young adult age-matched WKY rats. These results suggest that the immune response might play a more important role in the middle to late stages of the development of hypertension in SHRs.

Some studies have proposed that gut-derived, low-grade endotoxemia is a risk factor for cardiovascular events^29^. In gut dysbiosis, LPS, a component of gram-negative bacteria in the gut, enters the circulation due to dysfunction in the intestinal barrier scaffolding, leading to low-grade inflammation in several cell types, including endothelial cells and leukocytes. Activation of TLR4 signaling due to LPS causes systemic inflammation, including vascular endothelial dysfunction, by increasing the production of reactive oxidative stress and activating NF-κB signaling. Consistent with current hypotheses, a hypertensive model showed elevated plasma endotoxin levels with increased intestinal permeability^20^. In addition, TLR4 inhibition was shown to alleviate hypertension and vascular dysfunction in animal models^30, 31^. In our study, 10- to 15-week-old SHRs exhibited increased plasma levels of LPS along with increased intestinal permeability and vascular endothelial dysfunction. Importantly, treatment with *L. murinus* significantly reduced the LPS level in SHRs, suggesting that administration of probiotics improves the intestinal environment and therefore could be a candidate therapeutic approach for treating hypertension.

Mice with high salt intake reportedly exhibit a decrease in the abundance of *L. murinus* in feces. Treatment with *L. murinus* was shown to prevent salt-induced hypertension by modulating Th17 cells^32^. Additionally, high-salt challenge was shown to decrease *Lactobacillus* spp. in the feces of healthy individuals, although *Lactobacillus* is not a dominant microorganism in human feces. In the present study, we found a marked reduction in the abundance of *L. murinus* in 10- to 15-week-old SHRs compared with WKY rats. Treatment with *L. murinus* reversed the increase in blood pressure and vascular dysfunction and lowered plasma LPS levels.

As a preliminary study, we examined the gut microbiome profile in Japanese hypertensive patients. Analysis of 16S amplicons revealed that “*Lactobacillus* Low” was seen more in subjects with hypertension than “*Lactobacillus* High” (41.7% vs. 25.0%, Supplementary Fig. 14). Although this study was a preliminary data and there was no significance (p = 0.642, Fisher’s Exact Test with two-tailed), the results support a hypothesis that hypertension might be associated with decrease in members of the former *Lactobacillus* group species, including *Ligilactobacillus* spp. Because the etiology of hypertension is complex, the role of *Lactobacillus* group species in human clinical subjects warrants further investigation.

This study focused on determining whether the treatment to reverse a decrease in specific microbiota affects the gut and cardiovascular system using male hypertensive rats. We recognize that several evidences suggest that blood pressure response in females are different from males in hypertensive rats^33, 34^. Similarly, in addition to the effects of diet and age, it also has been shown sex differences in gut microbiota especially in animals^35^. Thus, future studies should be directed to the gender difference of cardiovascular response and gut microbiota in hypertension.

Several recent animal model studies^5^ and human clinical studies^6^ have revealed a link between gut dysbiosis and cardiovascular diseases, including hypertension. One feature of hypertension-related dysbiosis is an accompanying increase in intestinal permeability^8^. However, the pathophysiologic mechanism by which an imbalance in the gut microbiota causes hypertension remains unclear. In the present study, we demonstrated that treatment with *L. murinus* reverses the increase in blood pressure and vascular endothelial dysfunction in SHRs. In addition, treatment with *L. murinus* reversed the disruption in intestinal barrier function by up-regulating claudin 4 and down-regulating TNF-α expression, resulting in reduced plasma levels of LPS. Although we still did not observe any significant differences in members of the former *Lactobacillus* group, including *Ligilactobacillus* spp., in human subjects with hypertension compared with normotensive controls, our findings were consistent with the hypothesis that gut dysbiosis–mediated low-grade endotoxemia is an important risk factor for cardiovascular events^29^. These findings enhance our understanding of the potential role(s) of gut dysbiosis in the pathogenesis of hypertension and highlight the possibilities of a new class of therapeutic agents for treating hypertension.

## Methods

### Animals

Ten male Wistar Kyoto (WKY) rats (10–15 weeks old) and thirty male SHRs (10–15 weeks old) were used in this study, and SHRs were divided into three groups of ten rats each (no treatment, control and *L. murinus* treatment). In some analysis, the difference in the numbers of experiments was attributable to the amounts of samples available for the analysis. We have analyzed all the data obtained from appropriately collecting samples and conducting experiments to the extent possible. Care of these animals was in accordance with standards set forth by the National Institutes of Health guidelines for the care and use of experimental animals. All procedures were approved by the Animal Care and Use Committee of the Okayama University of Science (2021-086) and performed in accordance with ARRIVE guidelines.

All animals were acclimatized for at least 4 weeks at our animal facility. Animals were fed standard laboratory chow (CE-2) (CLEA Japan, Tokyo, Japan), water ad libitum and bred with standard laboratory bedding (CL-4163) (CLEA Japan, Tokyo, Japan). In some experiments, 10- to 13-week-old SHRs were randomized to receive 1 mL of 10% glycerol alone or 10% glycerol with *L. murinus* (as an equal mixture of WO strains described in the Results section, approximately 1 × 10^8^ CFU/mL per day) by oral gavage every day for 2 weeks. Animals were sacrificed with isoflurane overdose and exsanguination.

All animals were anesthetized with isoflurane (5%, MSD Animal Health, Readington Township, NJ) and euthanized by exsanguination at the end of the studies.

### 16S rDNA data analysis

Rats were acclimatized in the animal facility for 4–6 weeks, then fresh feces from WKY and SHR were collected. Fecal DNA was extracted by using commercial kit (NucleoBond HMW DNA, Takara, Japan) and DNA integrity was assessed using the 2200 TapeStation systems (Agilent Technologies, Santa Clara, CA, United States). Each sample corresponded to feces derived from one rat for the sequencing. These samples were prepared with LoopSeq 16S microbiome SSC 24plex kit (Loop Genomics, San Jose, CA, United States), sequenced on the illumine NextSeq and analyzed at Bioengineering Lab. Co, Ltd (Kanagawa, Japan). The datasets generated during the current study are available in the DDBJ Sequence Read Archive (DRA) repository (Accession number: DRA016499).

### Isolation and cultivation of *L. murinus*

Rats were acclimatized in the animal facility for 4–6 weeks, and then fresh feces were collected from WKY rats. Fecal samples were suspended in 0.9% NaCl and serially diluted tenfold to obtain 10^−3^, 10^−4^, 10^−5^ and 10^−6^ dilutions. Aliquots (100 μL) of the diluted solutions were spread on Rogosa agar plates (Merck, Rahway, NJ, USA) with or without 20 µg/mL vancomycin. The spread plates were incubated anaerobically at 37 °C for 48 h, after which single colonies were randomly picked and inoculated individually into MRS medium (Solabia Biokar Diagnostics, Ile-de-France, France). After incubation at 37 °C, each culture was collected to prepare glycerol stocks (final glycerol concentration adjusted to 25%). The glycerol stocks were stored at − 80 °C prior to use.

PCR using species-specific primer sets (57-76F and 202-182R) was performed to detect *L*. *murinus*^36^. Each 20-µL reaction mixture contained 1× GoTaq Green Master Mix (Promega), 0.5 µM each primer (57-76F and 202-182R), and 2 µL of the respective template DNA. PCRs were performed using the following temperature profiles: one cycle at 95 °C for 2 min, followed by 30 cycles at 95 °C for 30 s, 55 °C for 30 s, 72 °C for 45 s, and one cycle at 72 °C for 5 min. The resulting PCR amplicons were electrophoresed on 2% (w/v) agarose gel at a constant voltage of 100 V for 30 min at room temperature. The gel was then stained with ethidium bromide (0.5 µg/mL) and visualized under UV light at 312 nm.

Partial 16S rDNA sequences of the isolates in this study were amplified by PCR using the bacterial consensus primers Eu8f (*Escherichia coli* positions 8 to 27) and Eu1492r (*E*. *coli* positions 1510 to 1492)^37^. The sequences were determined and used for identification from the EZBioCloud database^38^. The 16S rDNA sequences of *L*. *murinus* isolates were registered in the DDBJ/ENA/GenBank under accession no. LC741556-LC741564.

Enterobacterial Repetitive Intergenic Consensus Polymerase Chain Reaction (ERIC-PCR) was conducted according to a method described previously, with a slight modification^39^. The 25-µL reaction mixture contained 1× DreamTaq Buffer including 3 mM MgCl_2_ (Thermo Scientific), 200 µM dNTP mix (Thermo Scientific), 1 µM each primer (ERIC1R and ERIC2), 0.625 U of DreamTaq DNA Polymerase (Thermo Scientific) and 25 ng of the respective template DNA. PCRs were performed using the following temperature profiles: one cycle at 95 °C for 3 min, followed by 35 cycles at 95 °C for 30 s, 48 °C for 1 min, 72 °C for 5 min, and one cycle at 72 °C for 7 min. The resulting PCR amplicons were electrophoresed on a 1.5% (w/v) agarose gel at a constant voltage of 70 V for 2 h 30 min at 4 °C. The gel was then stained with ethidium bromide (0.5 µg/mL) and visualized under UV light at 312 nm.

Bacterial strains used in this study were isolated from fresh feces of WKY rats (especially strains belonging to *L*. *murinus*), and *L*. *murinus* NBRC 14221 was used as the type strain. Isolates of *L*. *murinus* (WO strains described in the Results section) were incubated overnight at 37 °C in MRS medium. After harvesting, each culture of WO strains was equally mixed and washed with 0.9% NaCl. After centrifugation, the cell pellet was resuspended in 10% glycerol and used for oral administration of *L*. *murinus* to rats. The 10% glycerol solution containing *L*. *murinus* was divided into small portions and stored at − 80 °C prior to use.

Genomic DNA for PCR assays to detect *L*. *murinus* and 16S rDNA gene sequencing was extracted from fresh cells cultured in MRS medium by using PrepMan Ultra Sample Preparation Reagent (Applied Biosystems, Foster City, CA, USA). High-purity DNA was extracted for ERIC-PCR using the Wizard Genomic DNA Purification kit (Promega) following the manufacturer's protocol.

### Blood pressure measurement using a tail-cuff method

Systolic blood pressure was measured in Wistar-Kyoto rats (WKY) and spontaneously hypertensive rats (SHR) using a tail-cuff method (Softron, Tokyo, Japan) as previously described^40^. Rats were trained to reduce stress before measurement, and then blood pressure was measured at room temperature without a heater.

### Vascular function

Mesenteric arterial functions were assessed using a wire myograph preparation as previously described^41^. The secondary branches of mesenteric arteries were dissected and cut into small pieces. The preparations were suspended in an organ bath containing Kreb’s buffer (mmol/l: 118.3 NaCl, 4.7 KCl, 1.2 MgSO_4_, 1.2 KH_2_PO_4_, 25 NaHCO_3_, 2.5 CaCl_2_, and 11 glucose) maintained at 37 °C and 95% O_2_/5% CO_2_. Mesenteric arteries were then equilibrated for 45 min under a resting tension of 0.03–0.05 g, and contraction was recorded in response to KCl (10–100 mmol/L). Concentration-dependent response curves to acetylcholine (ACh, 0.3 nmol/L–30 μmol/L) or sodium nitroprusside (SNP, 0.1 nmol/L–10 μmol/L) were performed after an initial submaximal precontraction (50–70%) with U46619 (1–10 μmol/L). In addition, concentration-dependent response curves to serotonin (10 nmol/L–30 μmol/L) was performed.

### Measurement of intestinal permeability

Intestinal permeability in the ileum and proximal colon was assessed using the non-everted gut sac method, as previously described^42, 43^. Rats were euthanized after fasting for 12–14 h, and then the ileum or proximal colon was carefully dissected and cut into 3- to 5-cm pieces. FITC-Dextran, molecular weight 4 K or 70 K, was placed into the intestinal pieces and tied with cotton thread. The preparations were placed in an organ bath containing Tyrode’s solution without calcium (mmol/L: 136.9 NaCl, 2.68 KCl, 1.05 MgCl_2_, 0.41 NaH_2_PO_4_, 11.9 NaHCO_3_ and 5.55 glucose) and maintained at 37 °C and 95% O_2_/5% CO_2_. Medium was collected at 0, 30, 60, 90, and 120 min time points and analyzed for FITC on a fluorescent plate reader (Tecan, Männedorf, Switzerland). The apparent permeability of each individual intestinal sac was then calculated.

### Western blotting

Proximal colon and ileum were cleaned of fat and snap-frozen in liquid nitrogen. Frozen samples were homogenized in lysis buffer containing 50 mmol/l Tris·Cl buffer, 0.1 mmol/l EDTA (pH 7.5), 1% (wt/vol) Na deoxycholic acid, 1% (vol/vol) Nonidet P-40, and 0.1% (vol/vol) SDS with protease inhibitor and phosphatase inhibitors (Nacalai Tesque, Kyoto, Japan). Samples were rotated for 1 h at 4 °C and centrifuged (20,000*g*) for 10 min at 4 °C and then supernatants were collected. The protein concentration in the lysis buffer was determined by a Lowry assay (Nacalai Tesque). Equal amounts of proteins (50 μg) were separated by SDS-PAGE (8–12%) and transferred to a PVDF membrane (Millipore, Burlington, MA, USA). Membranes were blocked with 5% skim milk and incubated with primary antibodies at 4 °C overnight and then visualized using horseradish peroxidase-conjugated secondary antibodies (1:10,000 dilution, 1 h). Antibodies against claudin 4, occluding, cingulin, ZO-2 (Proteintech, Rosemont, IL, USA), and ZO-1 (Cell Signaling Technology, Danvers, MA, USA) were used for these experiments. GAPDH was used as a loading control (Santa Cruz, Dallas, TX, USA). Some membranes were cut prior to incubation with primary antibodies.

### Histology

Proximal colon was fixed with 10% neutral buffered formalin and subjected to paraffin sectioning. Sections were antigen retrieval in Tris–EDTA buffer (pH 9.0) and against claudin 4 (1:400 dilution, Proteintech, Rosemont, IL, USA). Intestinal morphology was determined by hematoxylin and eosin staining. Intestinal crypt and goblet cell were visualized by PAS staining. The area of the intestinal crypts stained with PAS and the number of goblet cells on intestinal mucosa were measured using ImageJ software (National Institutes of Health). When comparing between samples, adjustments to microscope settings (NIS-Elements, Nikon, Tokyo, Japan) were kept constant throughout image collection. The entire intestinal mucosa was captured and analyzed.

### Quantitative real-time RT-PCR (qPCR)

RNA was extracted from proximal colon, and quantitative real-time RT-PCR (qPCR) was performed as previously described^44^. Briefly, using a commercial kit (ReverTra Ace qPCR RT Master Mix, Toyobo, Osaka, Japan), cDNA was synthesized from 200–400 ng of total RNA extracted from the tissues using RNeasy spin columns (RNeasy Mini Kit, QIAGEN, Hilden, Germany). Each Q-PCR reaction was performed in duplicate. 2–5 ng of cDNA was subjected to gene expression assays using PrimeTime Gene Expression Master Mix (IDT) and the targeted probes. The following IDT probes were used on the Applied Biosystems QuantStudio System to evaluate gene expression levels: GAPDH (Rn.PT.39a.11180736.g), tumor necrosis factor α (TNF-α) (Rn.PT.58.36305979), IL-6 (Rn.PT.58.13840513), TGF-β (Rn.PT.58.6690138), LGR5 (Rn.PT.58.8656422), tert (Rn.PT.58.13068002), MUC2 (Rn.PT.58.45157067), MUC1 (Rn.PT.58.45226306), MUC4 (Rn.PT.58.44816706), TLR2 (Rn.PT.58.11978329), TLR4 (Rn.PT.58.11700071), CD3 (Rn.PT.58.13215743) and CD68 (Rn.PT.58.37733352). ΔΔCT was calculated using GAPDH as a reference gene to determine relative mRNA expression levels.

### Flow cytometry

Mesenteric lymph nodes and spleen were collected from rats. The tissues were mashed with slides in RPMI1640 medium and the solutions were filtered through a 100 μm cell strainer. After centrifugation, cells were resuspended with RPMI1640 medium containing 10% fetal bovine serum (FBS) and were adjusted to a concentration of 2 × 10^6^ cells/mL. A total of 1 mL of cells were seeded to each well on 24-well plate and incubated with 1 ug brefeldin A, 1 μg of ionomycin, and 50 ng of phorbol 12-myristate 13-acetate. After 4.5 h of incubation at 37 °C in 5% CO_2_, cells in each well were harvested, washed once with PBS, and blocked with anti-CD32 (BD biosciences, clone CD34-485, San Jose, CA, USA) for 10 min at 4 °C to avoid non-specific antibody binding to Fc-gamma receptors. After that, the cell surface staining was performed with mAbs anti-CD4 (Biolegend, clone W3/25), anti-CD45 (Biolegend, clone OX-1), and viability dye (LIVE/DIED^®^ Fixable Aqua Dead cell Stain Kit, ThermoFisher, Waltham, MA, USA) for 20 min at 4 °C. Then, cells were fixed with 4% paraformaldehyde at 4 °C for 20 min, washed once, and permeabilized by PBS containing 0.5% saponin (Nakalai tesque) and 0.5% bovine serum albumin (Fujifilm Wako, Richmond, VA, USA) at 4 °C for 30 min. After washing the fixed and permeabilized cells, intracellular staining was conducted with mAbs anti-IL17A (Biolegend, clone TC11-18H10.1), and anti-FoxP3 (eBiosciences, clone FJK-16s, San Diego, CA, USA), and anti-IFNγ (Biolegend, DB-1). Then, the cells were washed once, resuspended with PBS containing 2% FBS, and transferred to a 5 mL polystyrene tube through a 100 μm mesh. All samples were analyzed using a flow cytometer BD LSRFortessa (BD Biosciences) with FACSDIVA software.

### Plasma concentration of endotoxin

Plasma was collected from WKY rats and SHRs. The endotoxin level was measured using a commercial kit (Lonza, Basel, Switzerland).

### Human study

This study is approved by the Institutional Review Board of Ehime University Graduate School of Medicine (Reference no.1912011) and Institutional Review Board of the Okayama University of Science (Reference no. 3-2) and all methods in this study were performed in accordance with the relevant guidelines and regulations. Informed consent was obtained from all subjects by opt-out methods^45^. Briefly, Control (not taking anti-hypertensive medication) and Hypertensive (currently under hypertensive treatment or not taking anti-hypertensive medication) subjects were identified during their routine of health checkup at JA Ehime Kouseiren Checkup Center (Ehime, Japan). Major exclusion criteria for this study were as follows: (1) currently or previous antibiotic treatment within the last 2 months of study enrollment or (2) currently taking probiotics. Each subject was 50 years old or above of age. Upon registration with opt-out consent, an office ambient BP measurement was recorded for each individual and stool sample collection kits containing sample collection and return instructions were provided. Stool samples were collected using disposable test kit of bowel cancer by the patients, placed in a stool collection tube and fecal DNA was isolated. These samples were sent to Bioengineering Lab. Co, Ltd (Kanagawa, Japan), sequenced on the illumine MiSeq and analyzed by Qiime2. Data analysis was based on office BP measurements. Cutoff value for “Low” result of *Lactobacillus* was less than 0.047. Cutoff value was determined by receiver operating characteristic curve [ROC] analysis. χ2-tests were performed to find the significant differences between two groups. The datasets generated during the current study are available in the DDBJ Sequence Read Archive (DRA) repository (Accession number: DRA016495).

### Chemicals

ACh, SNP, 40 K and 70 K FITC-dextran and phorbol 12-myristate 13-acetate were purchased from Sigma (St. Louis, MO). KCl, serotonin, U46619 and ionomycin were obtained from Fujifilm Wako (Richmond, VA, USA).

### Statistical analysis

Experiments were performed in similar numbers between genotypes and control and treatment groups. Results are expressed as mean ± SEM. The data were statistically evaluated using GraphPad Prism software (San Diego, CA, USA). Where appropriate, the unpaired Student’s *t*-test was used to compare differences between two groups. In other experiments, two-way ANOVA (repeated measures when appropriate) with post hoc Tukey’s tests was used to compare data. Differences were considered significant at P < 0.05. Normality was assessed using a Kolmogorov–Smirnov test.

### Supplementary Information

Supplementary Figures.

## Data Availability

The data from this study are available from the corresponding author on reasonable request. The datasets of 16S rDNA data generated during the current study are available in the DDBJ Sequence Read Archive (DRA) repository (Accession number: DRA016499 and DRA016495).
