# Supplementary material for: Treatment with Ligilactobacillus murinus lowers blood pressure and intestinal permeability in spontaneously hypertensive rats
Source: Sci Rep. 2023 Sep 14;13:15197. doi: 10.1038/s41598-023-42377-7 (PMC10502128; doi:10.1038/s41598-023-42377-7)
Supplement: Supplementary file 1 — Supplementary Figures. [file 41598_2023_42377_MOESM1_ESM.pdf]

Online Supplement

Treatment with *Ligilactobacillus murinus* Lowers Blood Pressure and Intestinal Permeability in Spontaneously Hypertensive Rats

Masashi Mukohda*, Takanori Yano, Toshiyasu Matsui, Sho Nakamura, Jiro Miyamae, Kensuke Toyama, Ryoji Mitsui, Risuke Mizuno, Hiroshi Ozaki

Contents

Supplementary Figure S1-S14

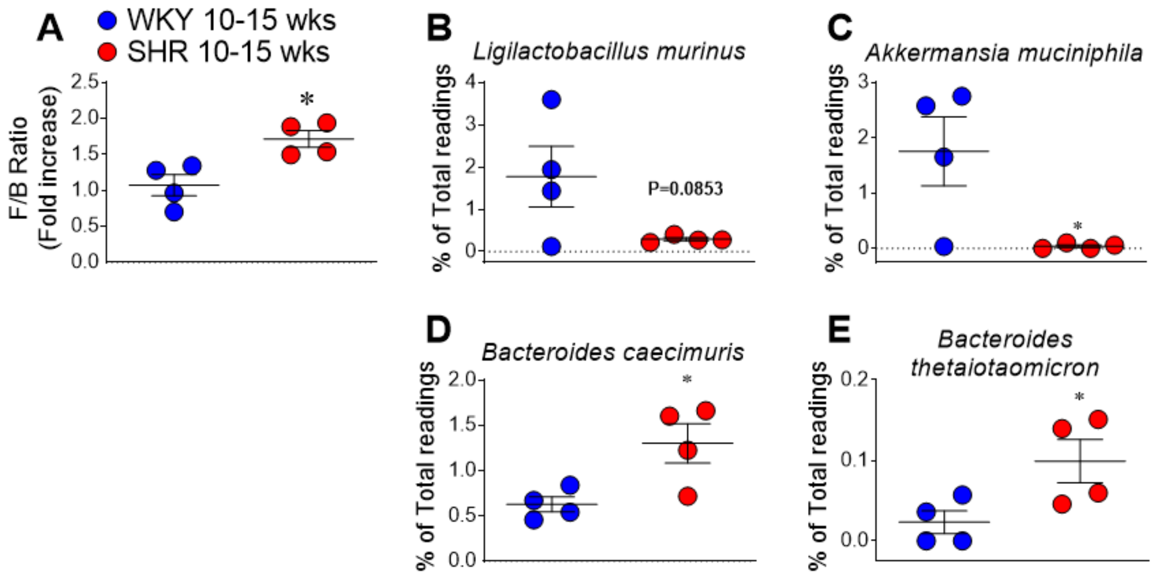

Supplementary Figure 1. *Altered gut microbiome in Wistar Kyoto rats (WKY) and spontaneously hypertensive rats (SHR)*

The microbial DNA from fecal samples of 10 to 15-week-old WKY and SHR were analyzed by 16S microbial amplicon sequencing (n=4 per group). All data are mean ± SEM. *P<0.05 vs. WKY. F/B ratio: Firmicutes/Bacteroidetes ratio.

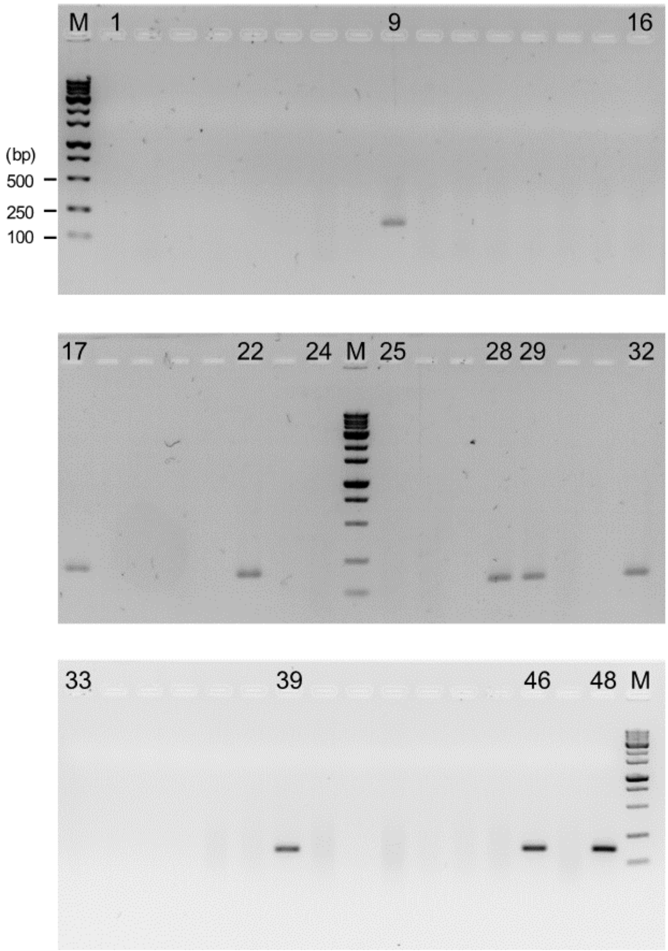

Supplementary Figure 2. *Detection of* Ligilactobacillus murinus (L. murinus) *by using species-specific PCR primers*

Target PCR product (146 bp) was obtained and found on the electrophoresis gel (nos. 9, 17, 22, 28, 29, 32, 39, 46 and 48). This indicates that nine strains of 48 candidates were detected as *L. murinus*. These isolates from feces of WKY were named *L. murinus* WO strains (WO9, WO17, WO22, WO28, WO29, WO32, WO39, WO46 and WO48). Each WO strain number corresponds to the detection number. M, Ladder marker.

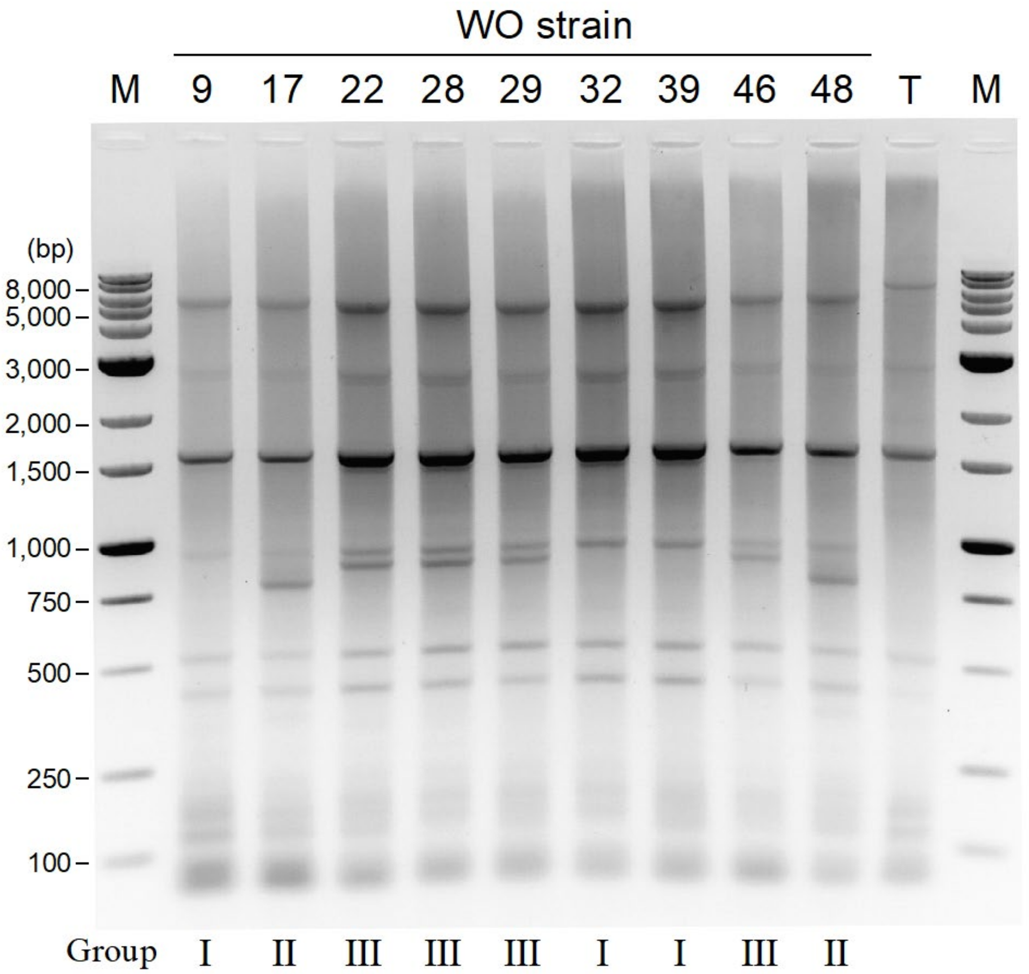

Supplementary Figure 3. *Detection of* L. murinus *by Enterobacterial Repetitive Intergenic Consensus Polymerase Chain Reaction (ERIC-PCR)*

The band patterns of ERIC-PCR showed that *L. murinus* WO strains are divided into three groups. Group I comprises WO9, WO32 and WO39. Group II consists of WO17 and WO48. Group III is comprised of WO22, WO28, WO29, and WO46. Strains WO9, WO17 and WO22 were isolated on Rogosa agar. Strains WO28, WO29, WO32, WO39, WO46 and WO48 were isolated on Rogosa agar with 20 μg/mL vancomycin. M, Ladder marker; T, *L. murinus* NBRC 14221 (type strain).

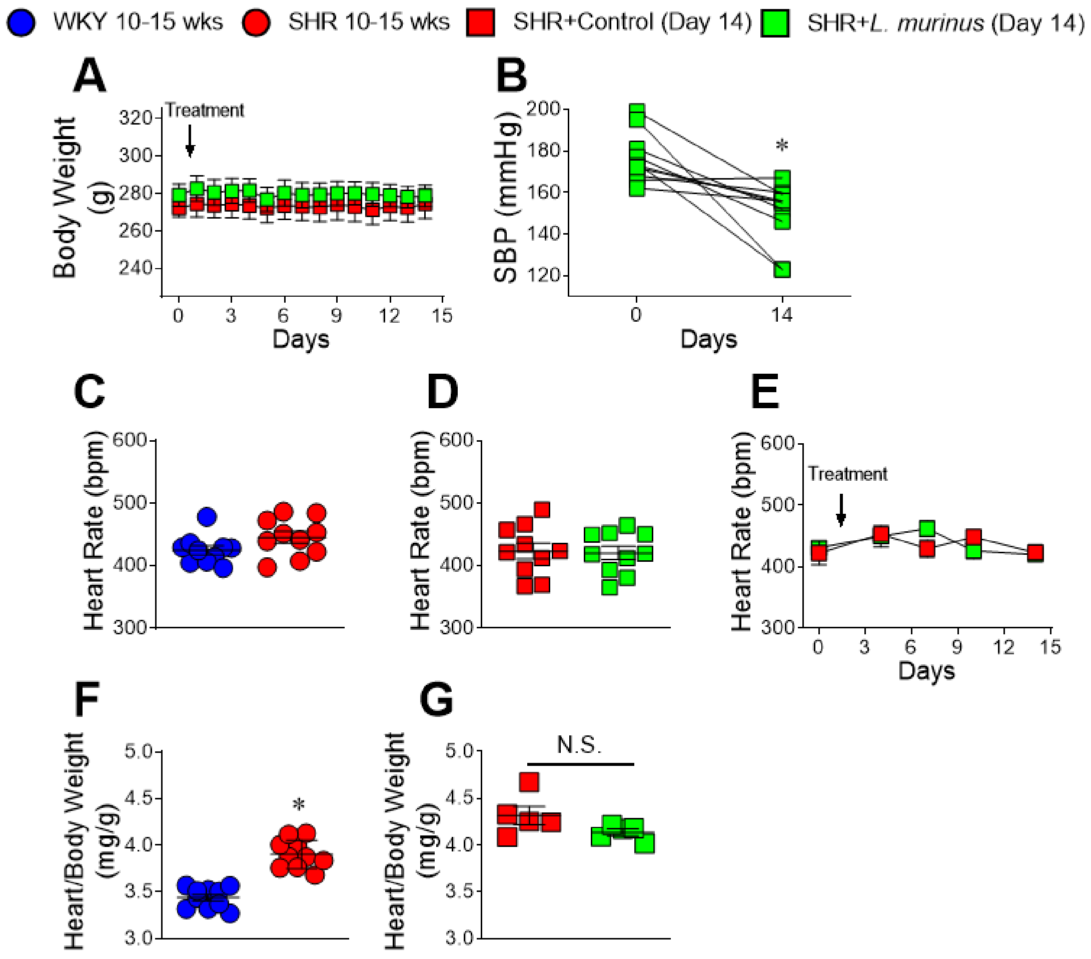

Supplementary Figure 4. *Body, heart rate and heart weight and systolic blood pressure (SBP) in SHR treated with* L. murinus

A) Body weight was measured for 14 days after initiation of Control or *L. murinus* treatment (arrow) in SHR (n=10 per group). B) SBP at day 0 (before) and 14 after treatment with *L. murinus* (n=10). C-E) Heart rate in 10- to 15-week-old WKY rats and SHR (C) (n=10 per group) or SHR+Control and SHR+*L. murinus* (D) (n=10 per group). Heart rate at day 0 (before) and 14 after treatment with *L. murinus* (E). F-G) Ratio of heart weight to body weight (HW/BW) in 10- to 15-week-old WKY rats and SHR (F) (n=10 per group) or SHR+Control and SHR+*L. murinus* (G) (n=5 per group). Data are mean ± SEM. **P*<0.05 vs. WKY.

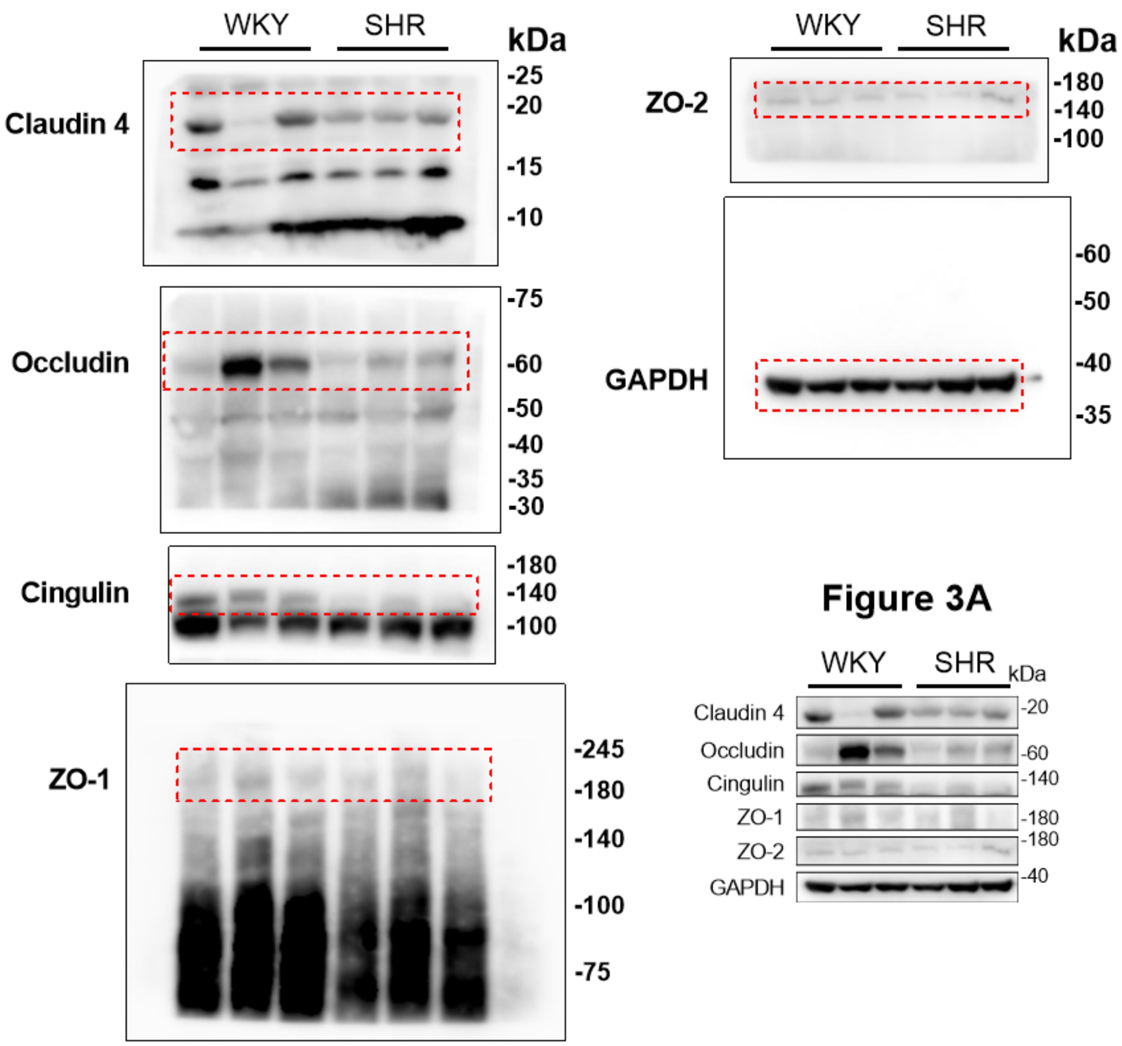

Supplementary Figure 5. *Original images for western blot in Figure 3A (WKY rats and SHRs)*

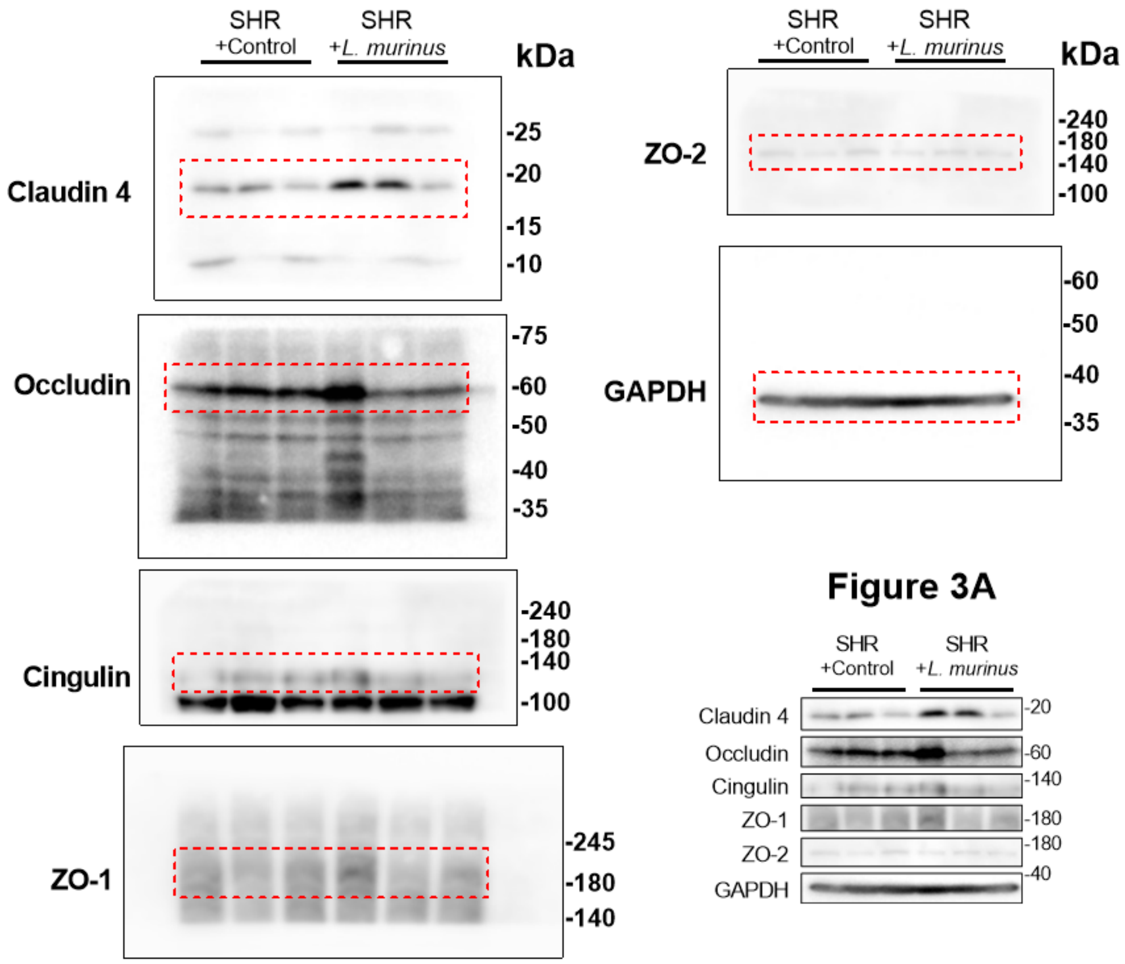

Supplementary Figure 6. *Original images for western blot in Figure 3A (SHR+Control and SHR+*L. murinus*)*

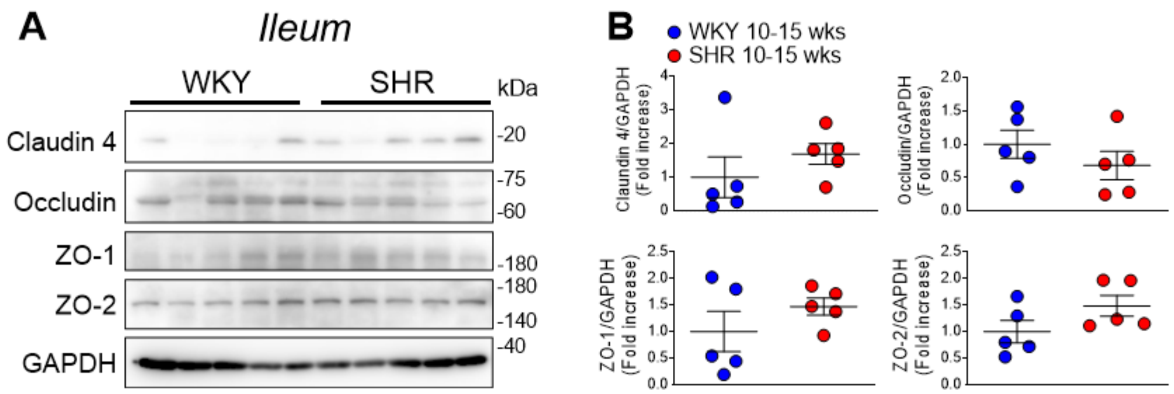

Supplementary Figure 7. *Expressions of tight junction proteins in ileum in SHR*

Western blot detecting the indicated proteins (representative of 5 experiments). Representative (A) and quantification (B) of western blots for claudin 4, occludin, ZO-1 and ZO-2 in ileum from 10- to 15-week old WKY and SHR (n=5 per group). All data are mean ± SEM.

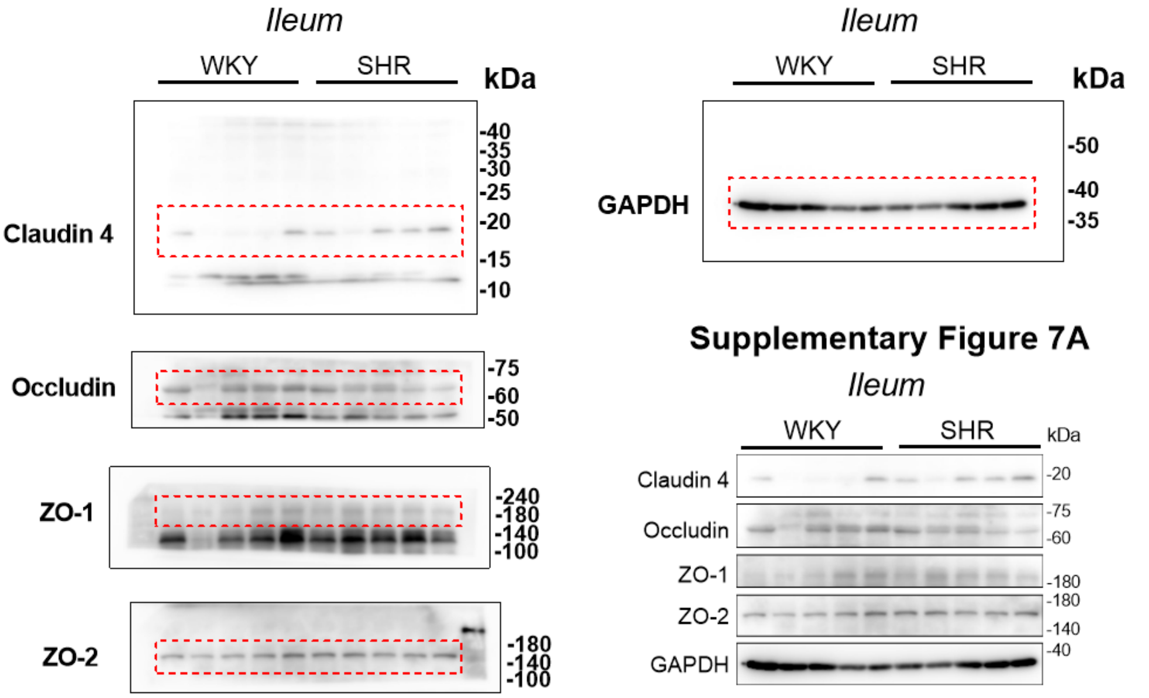

Supplementary Figure 8. *Original images for western blot in Supplemental Figure 7A*

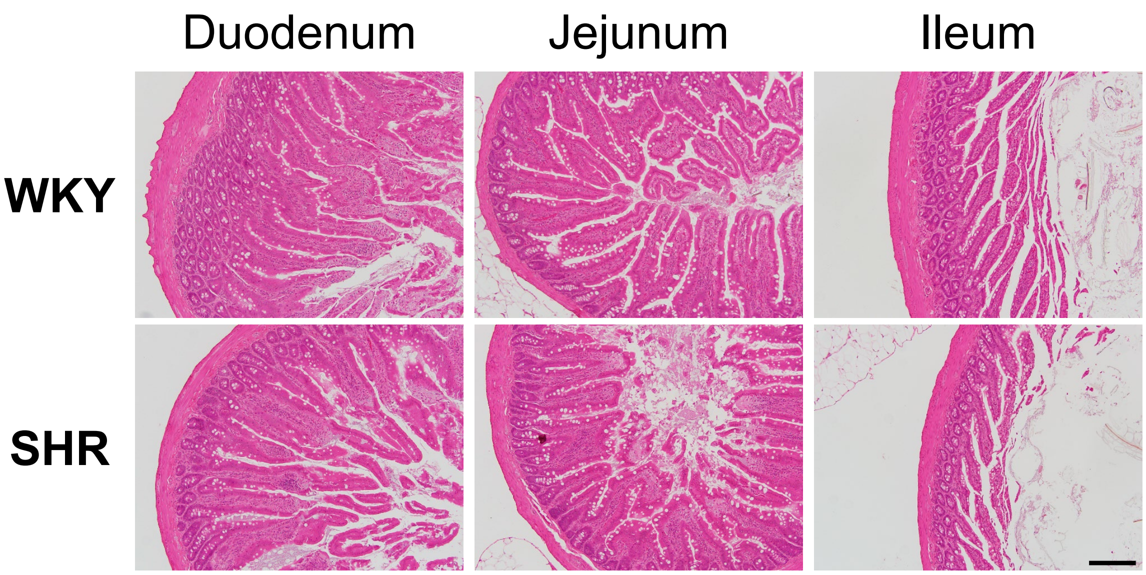

Supplementary Figure 9. *Intestinal morphological change in WKY and SHR*

Duodenum, jejunum, ileum and rectum were sectioned and stained with Hematoxylin and Eosin stain (H&E). Scale: 200 μm.

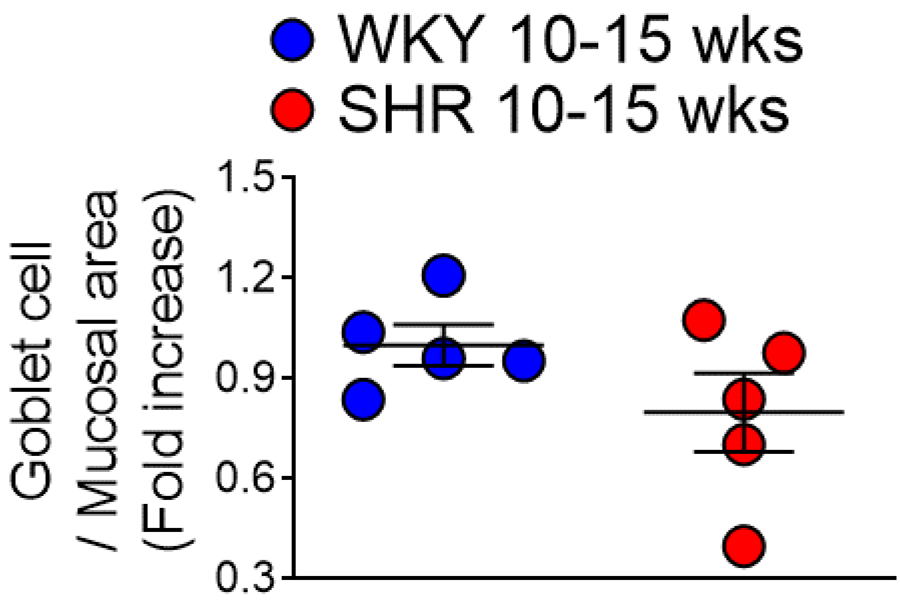

Supplementary Figure 10. *Goblet cells in proximal colon*

Number of goblet cells in mucosal area in proximal colon from 10- to 15-week old WKY and SHR were measured (n=5 per group). All data are mean ± SEM.

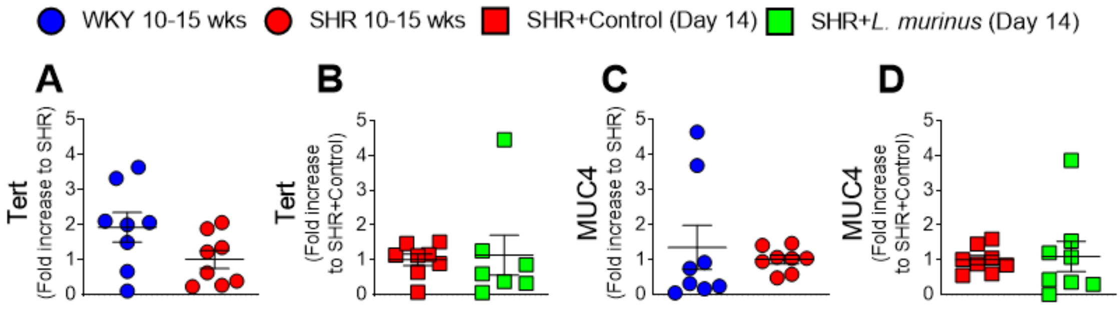

Supplementary Figure 11. *Markers of intestinal crypt in proximal colon from WKY, SHR and SHR treated with* L. murinus

Quantification of PCR for telomerase reverse transcriptase (tert) (A, B) and mucin 4 (C, D) in proximal colon from 10- to 15-week-old WKY and SHR (n=8 per group) or SHR+Control and SHR+*L. murinus* (n=7-8 per group). All data are mean ± SEM. **P*<0.05 vs. WKY.

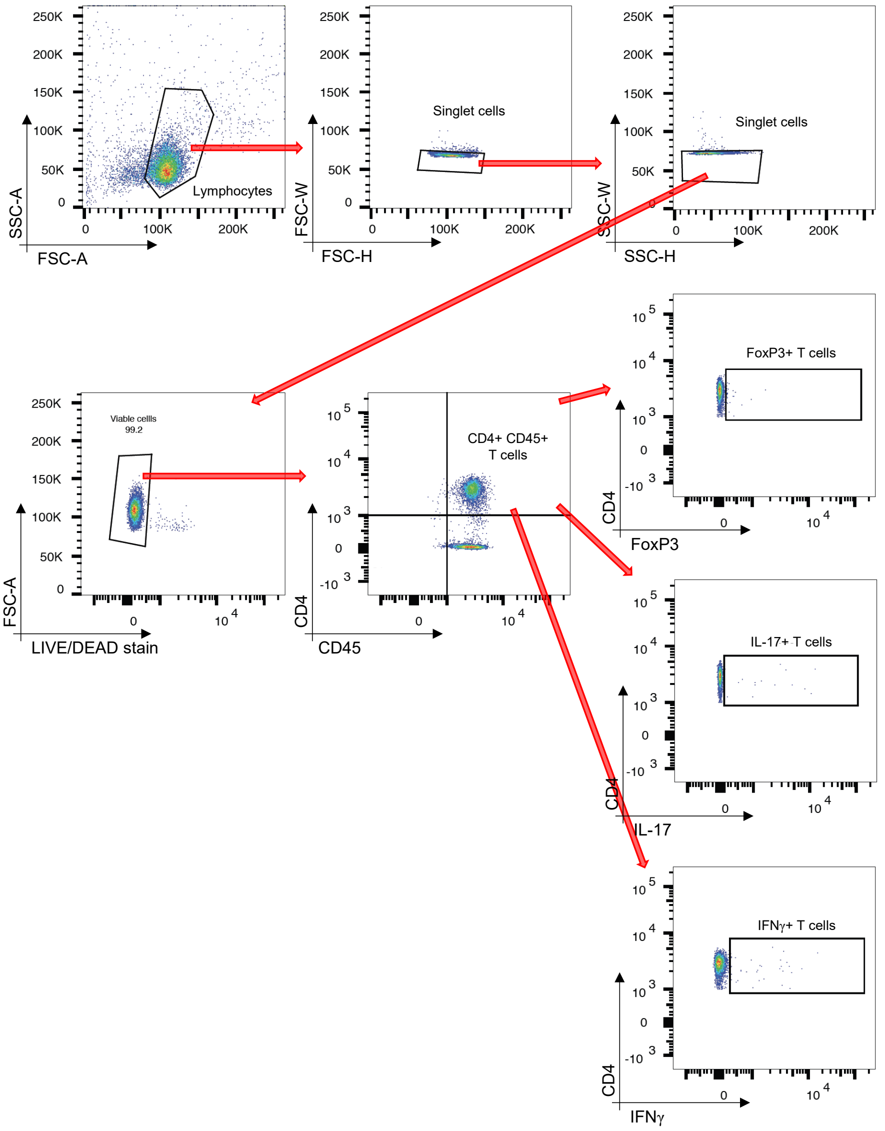

Supplementary Figure 12. *Gating strategy*

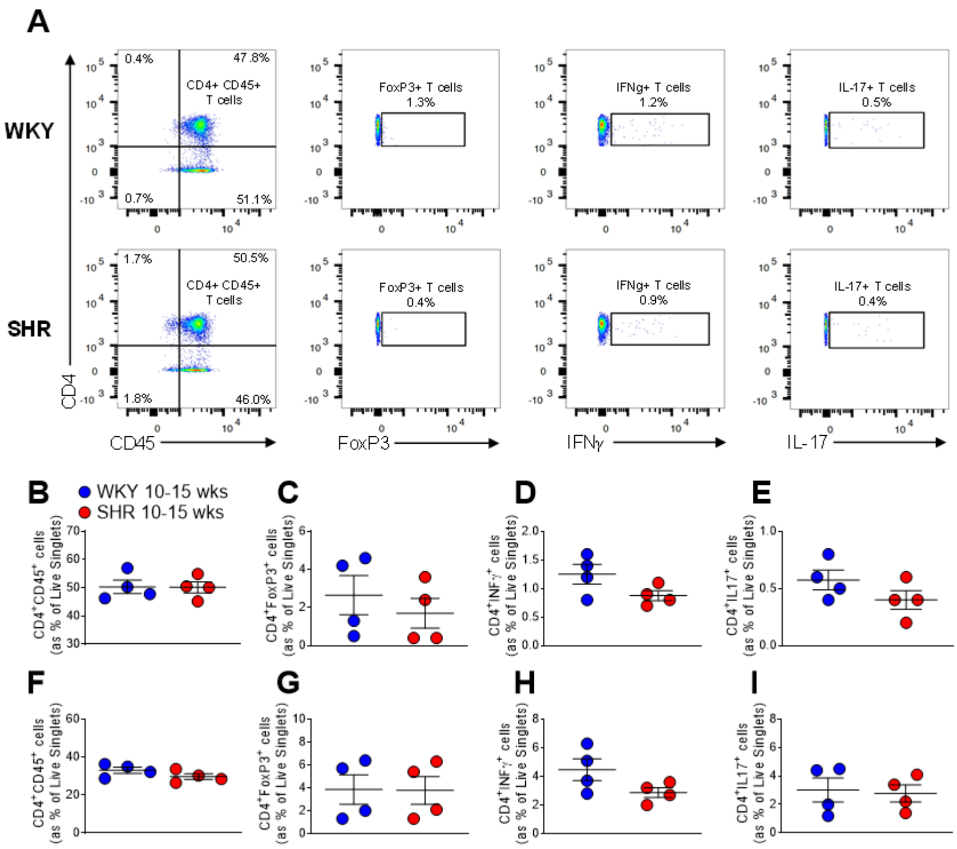

Supplementary Figure 13. *T cell polarization in mesenteric lymph nodes and spleen from WKY and SHR*

A) Flow cytometry analysis detecting the indicated immune cells (representative of 4 experiments). B-D) Quantification of flow cytometry for total T lymphocytes (CD4+ CD45+) (B), regulatory T cells (CD4+ FoxP3+) (C), T helper (Th) 1 (CD4+ INFγ+) (D) and Th17 (CD4+ IL-17a+) cells (E) were measured in mesenteric lymph nodes in 10-15 weeks old WKY and SHR (n=4 per group). F-I) Quantification of flow cytometry for total T lymphocytes (CD4+ CD45+) (F), regulatory T cells (CD4+ FoxP3+) (G), T helper (Th) 1 (CD4+ INFγ+) (H) and Th17 (CD4+ IL-17a+) cells (I) were measured in spleen in 10- 15 weeks old WKY and SHR (n=4 per group). All data are mean ± SEM.

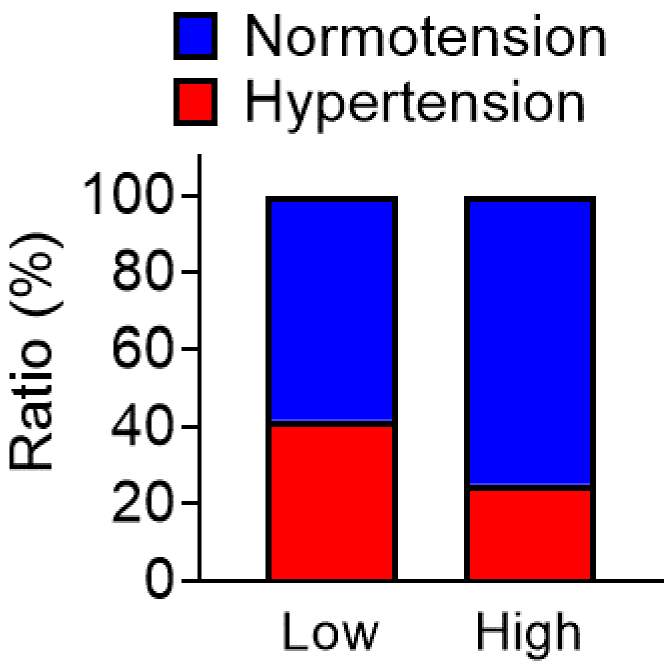

Supplementary Figure 14. *Fecal* Lactobacillus *in Hypertensive patient*

Ratio of *Lactobacillus* in subjects with normotensive controls (n=13) and hypertension (n=7).
